# Over Time, Do Anthropometric Measures Still Predict Diabetes Incidence in Chinese Han Nationality Population from Chengdu Community?

**DOI:** 10.1155/2013/239376

**Published:** 2013-10-12

**Authors:** Kai Liu, Sen He, Biying Hong, Rui Yang, Xiaoyan Zhou, Jiayue Feng, Si Wang, Xiaoping Chen

**Affiliations:** Department of Cardiovascular Medicine, West China Hospital, Sichuan University, Chengdu 610041, China

## Abstract

*Objective*. To examine whether anthropometric measures could predict diabetes incidence in a Chinese population during a 15-year follow-up. *Design and Methods*. The data were collected in 1992 and then again in 2007 from the same group of 687 individuals. Waist circumference, body mass index, waist to hip ratio, and waist to height ratio were collected based on a standard protocol. To assess the effects of baseline anthropometric measures on the new onset of diabetes, Cox's proportional hazards regression models were used to estimate the hazard ratios of them, and the discriminatory power of anthropometric measures for diabetes was assessed by the area under the receiver operating curve (AROC). *Results*. Seventy-four individuals were diagnosed with diabetes during a 15-year follow-up period (incidence: 10.8%). These anthropometric measures also predicted future diabetes during a long follow-up (*P* < 0.001). At 7-8 years, the AROC of central obesity measures (WC, WHpR, WHtR) were higher than that of general obesity measures (BMI) (*P* < 0.05). But, there were no significant differences among the four anthropometric measurements at 15 years. *Conclusions*. These anthropometric measures could still predict diabetes with a long time follow-up. However, the validity of anthropometric measures to predict incident diabetes may change with time.

## 1. Introduction

The prevalence of diabetes is high and increasing over the world. Especially in developing countries, the total number of adults with diabetes will increase 69% from 2010 to 2030 [1]. Diabetes mellitus (DM) prevention is recognized as a major public health priority in developing nations. Therefore, there is a great interest in identifying individuals at high risk of developing diabetes. Currently, it is well known that risk scoring models and anthropometric measures could predict future incidence of DM [2–8]. For example, the Finnish Diabetes Risk Score [9] and the Framingham Offspring Study simple clinical model [10], based on more than 10 years follow-up, have been used as a tool to predict the risk for diabetes. However, it seems that the predictive value of risk scoring models is time-varying. Stern et al. [11] published a clinical model to predict diabetes risk using fasting glucose and other routine clinical data, including age, sex, and ethnicity. This clinical model predicted 7.5-year incidence of diabetes better than the 2 h glucose load during oral glucose tolerance test in Mexican-American and non-Hispanic white participants from San Antonio Heart Study. McNeely et al. had found that the validity of this clinical model to predict incident diabetes was inconsistent between 5-6 years and 10 years in Japanese Americans aged less than 55 [12]. On the other hand, we do not know whether this phenomenon also exits in anthropometric measures, especially in Chinese Han nationality. To our knowledge, the duration of studies using anthropometric measures for predicting diabetes in Chinese Han nationality was mostly less than 15 years, and a little literature had been reported for more than 15 years. Therefore, we compared the predictive ability of anthropometric measures for predicting DM incidence based on a 15-year prospective study in Chinese adults.

## 2. Materials and Methods

### 2.1. Study Population

**Figure 1:**
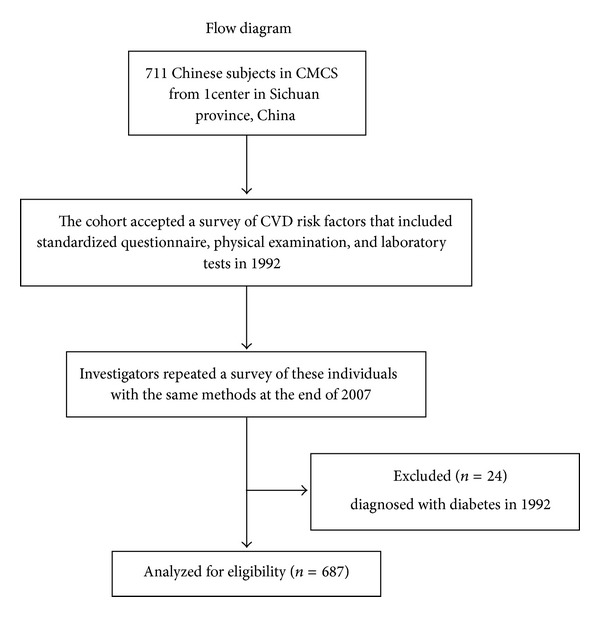
The flow chart of the study.

The study sample population was obtained from a Chinese Multi-provincial Cohort Study (CMCS) approved by the Beijing Institute of Heart, Lung and Blood Vessel Diseases for the entire duration, a nationwide, multicenter prospective cohort study on CVD risk factors in the Chinese. A total of 30121 Chinese subjects aged 35–64 and free of CVD at commencement was included by a multistage sampling method in which detailed information regarding the methodology had been previously reported [13–15]. Of these, 27003 subjects were recruited from 16 centers in 11 Chinese provinces between 1992 and 1993. At one of the centers (Sichuan, China), 711 individuals in an urban community located in Chengdu, Sichuan province, China, were included. The cohort also accepted a health examination in 2007 for a study supported by megaprojects of science research for the 11th five-year plan (trends in the incidence of metabolic syndrome and integrated control in China) with the same methods. Twenty-four individuals were excluded from this analysis for being diagnosed with diabetes in 1992. Therefore, 687 individuals with complete data were available (Figure 1).

### 2.2. Data Collection

In 1992, medicine doctors and nurses did a survey of cardiovascular disease (CVD) risk factors according to the Multinational Monitoring of trends and determinants in cardiovascular disease (MONICA) protocol [16]. Standardized questionnaire, physical examination, and laboratory tests were included in this survey.

#### 2.2.1. Questionnaire

All data collections were performed by specially trained doctors and nurses. A standardized questionnaire was used for collecting information on subjects' demographic characteristics, CVD risk factors, such as smoking status, alcohol consumption levels, physical activity, and family history of CVD.

#### 2.2.2. Physical Examination

Anthropometric measurements included height, weight, WC, and hip circumference measured while the patients were lightly clothed and without shoes after approximately 12 h of overnight fasting. Measurements were conducted using a calibrated weighing scale, wall-mounted stadiometer, and tape measure. The results were recorded to the nearest 0.2 kg, 1 cm, and 0.5 cm. Waist circumference (WC) was taken with the subject in a standing position, at the level midway between the lateral rib margin and the iliac crest. Hip circumference was measured at the level of the greater trochanter. BMI was calculated using the following formula: weight/height^2^ (kg/m^2^). WHpR was calculated as WC divided by hip circumference, and WHtR as WC divided by height. Blood pressure (BP) was measured in the right arm with subjects in a sitting position using a regular mercury sphygmomanometer after resting for 15 minutes. The three consecutive blood pressure readings were used for mean value of the BP.

#### 2.2.3. Laboratory Test

Fasting plasma glucose (FPG), fasting serum total cholesterol (TC), low-density lipoprotein cholesterol (LDL-C), high-density lipoprotein cholesterol (HDL-C), and triglyceride (TG) were included. Blood was drawn from the antecubital vein in the morning after 12-hour fasting. Biochemistry parameters were measured at the laboratory of West China Hospital (Chengdu, China). TC, TG, and FPG were all determined using the enzymatic method, while HDL-C was measured using the phosphotungstic acid/magnesium chloride precipitation method. The level of non-HDL cholesterol was calculated by subtracting HDL cholesterol from total cholesterol determined. The concentration of LDL-C was estimated using the Friedewald formula, and very low-density lipoprotein cholesterol was calculated by subtracting serum LDL-C and HDL-C from TC [13, 14, 17].

Detailed information about the variables used is available at http://www.ktl.fi/publications/monica/index.html.

In 2007, we repeated a survey of these participants with the same methods. This survey was approved by the Ministry of Health of China as well as the Ethics Committee of West China Hospital of Sichuan University. The study protocol conforms to the ethical guidelines of the 1975 Declaration of Helsinki as reflected in a priori approval by the institution's human research committee. All participants provided written informed consent.

### 2.3. Related Definitions

Hypertension was defined as having systolic blood pressure (SBP) of at least 140 mm Hg and/or diastolic blood pressure (DBP) of at least 90 mm Hg and/or currently taking antihypertensive medications. Diabetes mellitus was defined as one of the following at follow-up assessment: (1) fasting plasma glucose ≥ 7.0 mmol/L, (2) a positive response to the question, “Has a doctor ever told you that you have diabetes?”, or (3) current use of insulin or oral hypoglycemic agents. Smoking was defined as average cigarette consumption of at least 1 per day. Alcohol intake was defined as average intake of alcohol of at least 50 g/d. Physical activity was defined as exercise one or more times per week, at least 20 minutes for each time [15, 17, 18].

### 2.4. Statistical Analyses

Continuous variables were expressed as the mean ± SD or median (interquartile range) as appropriate. Differences of baseline characteristics between participants with and without diabetes were tested by independent *t*-test for normally distributed variables and by the nonparametric Mann-Whitney or Wilcoxon test for skewed variables. Categorical data were expressed as frequencies. Differences between participants with and without diabetes were tested by Chi-square test. This study population was stratified into quartiles of BMI, WC, WHpR, and WHtR. To assess the effects of baseline anthropometric measures on the new onset of diabetes, Cox's proportional hazards regression models were used to estimate the hazard ratios (HRs) of them, and the discriminatory power of anthropometric measures for diabetes was assessed by the area under the receiver operating curve (AROC). The hazard ratios (HRs) were computed for quartiles 2, 3, and 4 as compared with the lowest quartile in different Cox's proportional hazards regression models. Covariates including age, systolic blood pressure (SBP), TG, HDL-C, and FPG were fitted as continuous variables in the multivariate analyses and alcohol intake, smoking, regular physical exercise, and family history of diabetes were fitted as categorical variables. The point represented the largest sum of sensitivity and specificity on the receiver operating curve (ROC) was chosen to obtain these four measurements in predicting diabetes. The difference between AROCs was assessed using the algorithm developed by DeLong nonparametric approach. SPSS 13.0 and MedCalc 11.0 softwares were used. Statistical significance was defined as *P* < 0.05.

## 3. Result

### 3.1. Basic Characteristics of Subjects

687 eligible subjects studied at baseline completed the 7-8 years and the 15-year follow-up. The incidence of diabetes was 2.8% (*n* = 19) at 8 years and 10.8% (*n* = 74) at 15 year. The percentage of self-reported history was 67.6% (50/74) during the 15-year follow-up. Among the self-reported participants, 37 individuals were on antidiabetic drugs. For the other 24 diagnosed DM individuals, the average FPG was 9.5 mmol/L. The median was 9.1 mmol/L and the maximum and minimum were 16.2 mmol/L and 7.1 mmol/L, respectively. Characteristics of participants at baseline were shown in Table 1.Compared with subsequent nondiabetic subjects, the demographic data in 1992 showed that subsequent diabetic subjects had a higher BMI, WC, hip circumference, WHpR, WHtR, fasting plasma glucose, and triglycerides at 7-8 years and 15-year follow-up (all *P* < 0.001, Table 1).

### 3.2. Cox's Proportional Hazards Regression Models for Prediction of DM

**Figure 2:**
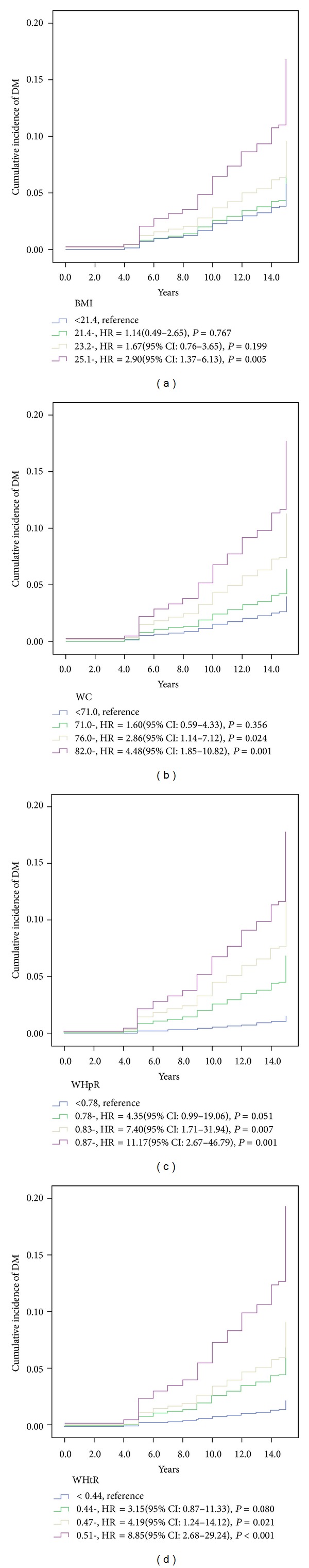
Cumulative incidence of DM in different anthropometric variables groups in multivariate COX-regression in Model 2. Model 2 adjusted for age, sex, smoking, alcohol intake, regular physical exercise, family history of diabetes, SBP, HDL, TG, and FPG. SBP = systolic blood pressure, HDL-C = high density lipoprotein cholesterol, TG = triglyceride, and FPG = fasting plasma glucose.

The univariate Cox's proportional hazards regression analysis presented that these anthropometric measures (BMI, WC, WHpR, and WHtR) could statistically increase the risk for the new onset of diabetes with a 15-year follow-up (Table 2). In the multivariate Cox's proportional hazards regression models, all measurements were significantly associated with risk of diabetes after adjustment for potential risk factors including age, sex, alcohol intake, smoking, regular physical exercise, family history of diabetes, and FPG. After further adjustment for SBP, TG, and HDL, the associations were reduced, but remained to be significant (Table 2). Time of diabetes onset was 11.2 ± 3.8 years.  Figure 2 showed the cumulative incidence of DM in different anthropometric variables groups in multivariate COX-regression in Model 2.

### 3.3. ROC Curves Analyses

The areas under the ROC curves were 0.668 (95% CI: 0.631–0.703) for BMI, 0.781 (95% CI: 0.748–0.811) for WC, 0.769 (0.736–0.800) for WHpR, and 0.768 (0.734–0.799) for WHtR at 7-8-year follow-up, respectively (all *P* < 0.05), while 0.668 (95% CI: 0.601–0.734) for BMI, 0.701 (95% CI: 0.641–0.760) for WC, 0.691 (95% CI: 0.637–0.748) for WHpR, and 0.715 (95% CI: 0.657–0.774) for WHtR (all *P* < 0.05, Table 3) at 15-year follow-up. Table 3 indicated that WC, WHpR, and WHtR were better than BMI for predicting incident diabetes at 7-8 years in Chinese Han nationality population from Chengdu community. However, the four anthropometric measurements had no significant difference for predicting 15-year incidence of diabetes.

## 4. Discussion

The goal of the study was to assess whether BMI, WC, WHpR, and WHtR could predict future diabetes on the basis of data collected from a general Chinese group during 15-year follow-up and to assess whether the validity of anthropometric measures to predict incident diabetes were stable as time goes by, especially in Chinese Han nationality. After adjusting for potential confounders, our findings showed that both general obesity measures (BMI) and central obesity measures (WC, WHpR, WHtR) could predict future diabetes in a Chinese population after a long follow up. On the other hand, WC, WHpR, and WHtR were better than BMI for predicting incident diabetes at 7-8 years in Chinese Han nationality population from Chengdu community. However, the four anthropometric measurements had not significant difference for predicting 15-year incidence of diabetes.

In recent years, some studies showed a more close association between diabetes and central obesity measures than BMI [19–23], whereas other studies showed no significant differences between the obesity measures [24–26]. Meanwhile, meta-analysis studies also showed inconsistent results [27, 28]. However, we reviewed literatures of previous prospective studies comparing BMI, WC, WHpR, and WHtR for predicting incidence of diabetes and found that many studies reporting a difference between the obesity measures did not use appropriate study statistical methods or that it did not investigate all indicators with data on only 1 or more of the 4 indicators (i.e., BMI, WC, and WHpR) with regard to the association with diabetes risk. More importantly, length of the follow-up periods might influence study results [29–46] (See Supplementary Table 1 available online at http://dx.doi.org/10.1155/2013/239376). Firstly, we compared the predictive ability of the four anthropometric measures for predicting DM incidence based on a 15-year prospective study in Chinese adults. Similar to our findings, in 6–10 years and 11–15 years longitudinal follow-up groups in Supplementary Table 1, the majority of trials had shown no significant difference between obesity measures. Nyamdorj et al. thought that different methods of collecting numbers of onset diabetes and obesity measures might result in this result [36]. For example, Folsom et al. [44] and Wang et al. [45] had shown that anthropometric indicators were equally associated with diabetes. Diabetes incidence was self-reported and anthropometric indicators were self-measured by the participants in the two studies. But, investigators from the San Antonio Heart Study [39] and EPIC-Potsdam Study [38], with similar methods, had demonstrated higher risk with WC or WHpR than BMI in predicting diabetes incidence. Our findings revealed that WC, WHpR, and WHtR were better than BMI for predicting incident diabetes at 7-8 years in the same population. However, the four indictors did not have a significant difference in our study at 15 years. This prompted that our different follow-up time may affect the results. A study reported that over 10 years, total fat mass and fat mass except subcutaneous fat increased, whereas body weight did not change [47]. It was possible that many subjects tend to become abdominally obese rather than become totally obese during follow-up, and the impact of baseline body fat distribution on diabetes risk was weakened with lengthening of the follow-up period [28].

On the other hand, racial and regional differences in the association between obesity and the risk for diabetes might also have influenced the results. Although the prevalence of obesity is much lower in Asia than in Western countries, the prevalence of diabetes is similar between the two regions [48], and diabetes occurs in Asians who are less obese [49]. Therefore, we reviewed studies that compared anthropometric measures in predicting risk of diabetes in Chinese mainland [50–53] (Supplementary Table 2). We needed to know that most of the studies were cross-sectional. There might be a greater bias risk in data collection (i.e., potential detection bias), and the cross-sectional data showed only the association with present risk factor conditions but did not directly predict the future risk of DM. This was the advantage of our study. A long follow-up time was to avoid not only insufficiency of new onset diabetes cases in short-term follow-up that underestimated the predictive power of the measurement indexes but also potential detection bias in cross-sectional studies that exaggerate the statistical power of the indexes. Therefore, more longitudinal studies are required to further investigate the issue. Taking into account the sample size, sex, and region, we cannot extend our findings to general population. But we need to know that the length of follow-up period is a factor that cannot be ignored.

Another advantage was that a standardized protocol was used and all anthropometric variables were collected using direct measurement by trained doctors and nurses rather than self-report. This may increase the accuracy of the association between anthropometric measures with diabetes risk. In contrast to these advantages, there were some limitations of our study. The first was that this study had relatively small sample size. This limited the study, and we could not analyze the association between anthropometric measures with diabetes risk classified by gender. The results of our study may have limited statistical power; but we still can get some clues. The second was the way to diagnose diabetes mellitus. During the investigation, taken into account the financing and feasibility, we had diagnosed diabetes by measuring fasting plasma glucose and patient self-reports rather than oral glucose tolerance test means. Some individuals would have developed DM that was not detectable by changes in fasting glucose alone or by clinical history, leading to the underestimation of the experimental results. The third was that all the participants came from Chengdu province, China. We cannot extend our findings to general population.

In summary, our findings showed that anthropometric measures could predict diabetes with a long-time follow-up. However, the validity of anthropometric measures to predict incident diabetes may change with time. Future researches may be warranted to assess whether this phenomenon still exists in mainland China with different duration of follow-up time in a large population.

## Supplementary Material

Supplementary Table 1: Comparison of anthropometric measures in predicting risk of diabetes from different prospective studies.
Supplementary Table 2: Comparison of anthropometric measures in predicting risk of diabetes from different studies in Chinese mainland.

## Figures and Tables

**Table 1:** Baseline characteristics of the population according to diabetes status at follow-up at 7-8 and 15 years.

Variable	Diabetes status at 7-8 year-follow-up			Diabetes status at 15-year follow-up		
	Subsequent diabetic patients (*n* = 19)	Subsequent nondiabetic patients (*n* = 668)	*P* value	Subsequent diabetic patients (*n* = 74)	Subsequent nondiabetic patients (*n* = 613)	*P* value
Years	50.6 ± 6.6	48.1 ± 6.2	0.08	49.8 ± 5.7	47.9 ± 6.2	0.013
Sex (male)	12 (63.1)	387 (57.9)	0.65	48 (64.9)	351 (57.3)	0.21
BMI (kg/m^2^)	25.4 ± 3.6	23.3 ± 2.8	0.002	25.1 ± 3.3	23.2 ± 2.6	<0.001
SBP (mmHg)	119.1 ± 18.7	114.4 ± 15.2	0.19	118.9 ± 18.2	114.0 ± 14.9	0.021
DBP (mmHg)	75.8 ± 10.1	73.6 ± 9.1	0.3	75.7 ± 9.6	73.4 ± 9.0	0.095
FPG (mmol/L)	4.7 ± 0.8	4.3 ± 0.7	0.007	4.6 ± 0.8	4.2 ± 0.7	<0.001
TC (mmol/L)	4.6 ± 0.7	4.5 ± 0.8	0.38	4.7 ± 0.7	4.5 ± 0.8	0.023
TG (mmol/L)	2.97 ± 1.51	2.08 ± 0.96	<0.001	2.6 ± 1.2	2.1 ± 0.9	<0.001
LDL-C (mmol/L)	2.0 ± 1.0	2.3 ± 0.8	0.12	2.3 ± 0.9	2.3 ± 0.8	0.776
HDL-C (mmol/L)	1.29 ± 0.33	1.24 ± 0.23	0.41	1.18 ± 0.24	1.25 ± 0.24	0.007
Waist to hip ratio	0.88 ± 0.05	0.83 ± 0.06	<0.001	0.86 ± 0.05	0.83 ± 0.06	<0.001
Waist to height ratio	0.52 ± 0.04	0.47 ± 0.05	<0.001	0.51 ± 0.05	0.47 ± 0.05	<0.001
Waist (cm)	85.4 ± 9.1	76.3 ± 7.7	<0.001	82.0 ± 8.4	75.9 ± 7.6	<0.001
Hip (cm)	96.6 ± 8.2	92.1 ± 5.7	0.001	94.9 ± 7.1	91.9 ± 5.6	<0.001
Smoking	8 (42.1)	240 (35.9)	0.58	32 (43.2)	216 (35.2)	0.176
Alcohol intake	3 (15.8)	84 (12.6)	0.68	12 (16.2)	75 (12.2)	0.331
Physical activity	2 (10.5)	144 (21.6)	0.25	14 (18.9)	132 (21.5)	0.604
Hypertension	3 (15.8)	101 (15.1)	0.94	16 (21.6)	88 (14.4)	0.099
Family history of diabetes	4 (21.1)	22 (3.3)	<0.001	6 (8.1)	20 (3.3)	0.039

Data are means ± SD or *n* (%).

**Table 2:** Univariate and multivariate COX-regression models for prediction of diabetes in different models.

Variable	Cases	Univariate regression		Model 1		Model 2	
		HR (95% CI)	*P* value	HR (95% CI)	*P* value	HR (95% CI)	*P* value
*Quartiles *							
BMI (kg/m^2^)							
	167	1.00	NA	1.00	NA	1.00	NA
21.4-	167	1.19 (0.52–2.76)	0.679	1.20 (0.51–2.78)	0.680	1.14 (0.49–2.65)	0.767
23.2-	174	1.78 (0.82–3.85)	0.145	1.80 (0.83–3.92)	0.140	1.67 (0.76–3.65)	0.199
25.1-	179	3.43 (1.69–6.94)	0.001	3.53 (1.72–7.25)	0.001	2.90 (1.37–6.13)	0.005
* P *for trends			<0.001		<0.001		0.008
WC (cm)							
	163	1.00	NA	1.00	NA	1.00	NA
71.0-	171	1.75 (0.65–4.73)	0.271	1.61 (0.60–4.37)	0.348	1.60 (0.59–4.33)	0.356
76.0-	166	3.55 (1.43–8.79)	0.006	3.05 (1.23–7.60)	0.017	2.86 (1.14–7.12)	0.024
82.0-	187	5.72 (2.41–13.58)	<0.001	5.23 (2.20–12.44)	<0.001	4.48 (1.85–10.82)	0.001
* P *for trends			<0.001		<0.001		0.001
WHpR							
	132	1.00	NA	1.00	NA	1.00	NA
0.78-	203	4.99 (1.14–21.82)	0.033	4.63 (1.06–20.27)	0.042	4.35 (0.99–19.06)	0.051
0.83-	153	8.93 (2.09–38.18)	0.003	8.10 (1.89–34.84)	0.005	7.40 (1.71–31.94)	0.007
0.87-	199	13.37 (3.22–55.48)	<0.001	13.13 (3.16–54.49)	<0.001	11.17 (2.67–46.79)	0.001
* P *for trends			<0.001		<0.001		<0.001
WHtR							
	140	1.00	NA	1.00	NA	1.00	NA
0.44-	143	3.71 (1.03–13.29)	0.044	3.35 (0.93–12.04)	0.064	3.15 (0.87–11.33)	0.080
0.47-	225	4.74 (1.42–15.85)	0.011	4.59 (1.37–15.4)	0.014	4.19 (1.24–14.12)	0.021
0.51-	179	10.97 (3.39–35.52)	<0.001	10.47 (3.22–34.07)	<0.001	8.85 (2.68–29.24)	<0.001
* P *for trends			<0.001		<0.001		<0.001

Model 1: adjusted for age, sex, smoking, alcohol intake, regular physical exercise, family history of diabetes, and FPG.

Model 2: adjusted for age, sex, smoking, alcohol intake, regular physical exercise, family history of diabetes, SBP, HDL, TG, and FPG.

**Table 3:** Areas under the ROC curve for various anthropometric measurements used to predict diabetes incidence.

Variables	Diabetes incidence after 7-8 years					*P* value compared to BMI	Diabetes incidence after 15 years					*P* value compared to BMI
	AUC (95% CI)	*P* value	Sensitivity	Specificity	ΔAUC (95% CI)		AUC (95% CI)	*P* value	Sensitivity	Specificity	ΔAUC (95% CI)	
BMI	0.668 (0.631–0.703)	0.026	0.579	0.796	—	—	0.668 (0.601–0.734)	<0.001	0.581	0.697	—	—
WC	0.781 (0.748–0.811)	<0.001	0.684	0.783	0.113 (0.0291–0.197)^§^	0.008	0.701 (0.641–0.760)	<0.001	0.851	0.44	0.033 (−0.018–0.084)^§^	0.205
WHpR	0.769 (0.736–0.800)	<0.001	0.684	0.835	0.101 (−0.047–0.251)^§^	0.183	0.691 (0.637–0.748)	<0.001	0.865	0.452	0.023 (−0.055–0.101)^§^	0.558
WHtR	0.768 (0.734–0.799)	<0.001	0.684	0.783	0.099 (0.027–0.173)^§^	0.007	0.715 (0.657–0.774)	<0.001	0.77	0.548	0.047 (0.003–0.091)^§^	0.036

ΔAUC: difference between areas.

^§^Compare to BMI.
